# Predictive value of estimated pulse wave velocity for cardiovascular and all-cause mortality in individuals with obesity

**DOI:** 10.1186/s13098-023-01011-2

**Published:** 2023-03-09

**Authors:** Daidi Li, Feng Cao, Wenke Cheng, Yanyan Xu, Chuang Yang

**Affiliations:** 1 Blood Purification Center, Bengbu Third People’s Hospital, Bengfu, 233000 China; 2 Department of General, Visceral and Transplantation Surgery, University Hospital RWTH Aachen, 52074 Aachen, Germany; 3 Medical Faculty, University of Leipzig, Leipzig, Germany; 4 Department of General Surgery, The Second Hospital of Anhui Medical University, Hefei, China; 5 Department of Visceral, Transplant, Thoracic and Vascular Surgery, University Hospital Leipzig, Leipzig, Germany

**Keywords:** Cardiovascular, Estimated pulse wave velocity, Mortality, Obesity

## Abstract

**Background:**

Estimated pulse wave velocity (ePWV) has revealed excellent performance in predicting cardiovascular disease (CVD) risk. However, whether ePWV predicts all-cause mortality and CVD mortality in populations with obesity remains elusive.

**Methods:**

We performed a prospective cohort including 49,116 participants from the National Health and Nutrition Examination Survey from 2005 to 2014. Arterial stiffness was evaluated by ePWV. Weighted univariate, multivariate Cox regression and receiver operating characteristic curve (ROC) analysis was used to assess the effects of ePWV on the risk of all-cause and CVD mortality. In addition, the two-piecewise linear regression analysis was used to describe the trend of ePWV affecting mortality and identify the thresholds that significantly affect mortality.

**Results:**

A total of 9929 participants with obesity with ePWV data and 833 deaths were enrolled. Based on the multivariate Cox regression results, the high ePWV group had a 1.25-fold higher risk of all-cause mortality and a 5.76-fold higher risk of CVD mortality than the low-ePWV group. All-cause and CVD mortality risk increased by 123% and 44%, respectively, for every 1 m/s increase in ePWV. ROC results showed that ePWV had an excellent accuracy in predicting all-cause mortality (AUC = 0.801) and CVD mortality (AUC = 0.806). Furthermore, the two-piecewise linear regression analysis exhibited that the minimal threshold at which ePWV affected participant mortality was 6.7 m/s for all-cause mortality and 7.2 m/s for CVD mortality.

**Conclusions:**

ePWV was an independent risk factor for mortality in populations with obesity. High ePWV levels were associated with an increased all-cause and CVD mortality. Thus, ePWV can be considered a novel biomarker to assess mortality risk in patients with obesity.

**Supplementary Information:**

The online version contains supplementary material available at 10.1186/s13098-023-01011-2.

## Introduction

Obesity (BMI ≥ 30 kg/m^2^) is the excessive accumulation or abnormal distribution of body fat and is closely related to metabolic disorders which can be divided into class I obesity (BMI, 30.0–34.9 kg/m^2^), class II obesity (BMI, 35.0–39.9 kg/m^2^) and class III obesity(BMI ≥ 40 kg/m^2^) based on BMI [1]. At present, obesity is a growing global public health issue that affects both adults and children [2, 3]. Since nutrient metabolism is essential for the survival, growth and development of all organisms, obesity is associated with a wide range of diseases including dyslipidemia, cardiovascular diseases (CVD), type 2 diabetes mellitus, hypertension, hepatic steatosis, stroke, gallbladder diseases, osteoarthritis and sleep apnea, increasing the risk of mortality for patients with obesity [4]. Hence, it has crucial to identify clinical indicators linked to mortality caused by obesity or obesity-related complications.

Arterial stiffness plays a crucial role in the development of CVD and is strongly associated with mortality in populations with obesity [5]. Pulse wave velocity (PWV) is reported to be a novel non-invasive measurement of artery stiffness [6]. Several methods of measuring PWV have emerged over the past several years, such as carotid-femoral PWV (cfPWV) and brachial-ankle PWV (baPWV), of which cfPWV has been considered the standard method used to measure artery stiffness [7]. Although both cfPWV and baPWV have standardized measurement procedures [8], they require specialized and expensive devices that are rarely available in a primary hospital, thus, limiting their application in population screening [9]. To address this issue, researchers used estimated pulse wave velocity (ePWV) to assess the degree of aortic stiffness using an algorithm including age and mean blood pressure (MBP) [10]. At present, ePWV is considered an alternative to cfPWV [11], which may have critical importance in forecasting morbidity and mortality of people with obesity with different complications [12]. In this study, we analyzed the correlation between e-PWV and mortality in patients with obesity.

## Methods

### Study design and population

This was a prospective cohort with data from the National Health and Nutrition Examination Survey (NHANES) from 2005 to 2014, with follow-up till December 31, 2019. The data comprised interviews, home or mobile physical examinations and laboratory tests. It followed a complex, stratified and multi-stage probability design concept and was audited and managed by the National Centre for Health Statistics. The survey was performed every 2 years; all participants signed informed consent; and further specific information, sampling methods and data collection procedures can be obtained here [13].

Data from a total of 49,116 participants were collected in five cycles from NHANES between 2005 and 2014 (2 years/cycle), and 9929 participants with obesity (BMI ≥ 30 kg/m^2^)[1] were identified. We excluded 859 participants who were diagnosed with cancer, 186 participants who were pregnant, and 456 without ePWV data. Further, we excluded 113 participants who died within 2 years of follow-up for reducing the potential reverse causation bias; thus, 8315 participants were eventually included in this study. Elaborated information is available at https://wwwn.cdc.gov/Nchs/Nhanes/.

### Measurement of ePWV

ePWV was calculated using the following algorithm [14]: ePWV = 9.587–0.402 * age + 4.560 * 10^–3^ * age^2^ -2.621 * 10^–5^ * age^2^ * MBP + 3.176 * 10^–3^ * MBP * age—1.832 * 10^–2^ * MBP.

In this algorithm, age was measured in years, and MBP was calculated as diastolic BP (DBP) + 0.4 * [systolic BP -DBP]. Participants’ BP was measured after 5 min of quiet rest. These operations were conducted by NHANES technicians. The average of at least three measurements was used as the BP value.

### Study endpoints

The main outcomes in this study included CVD and all-cause mortality. All-cause mortality was the sum of all deaths whereas CVD mortality was diagnosed per the International Classification of Diseases version 10 codes (ICD-10 I00-I09, I11, I13 or I20-I51).

### Covariates

We collected and categorized covariates such as age (≤ 60 years and > 60 years), sex (male/female), race (non-Hispanic white people, non-Hispanic Black people, Mexican Americans, etc.), educational level (less than grade 9, 9 − 11 grade/graduated from high school or equivalent and college graduated or above), marriage (unmarried, married, separated, divorced, widowed and those living with partner/others), family income, smoking and drinking status. The smoking status was classified into the following: Never smoked (< 100 cigarettes/session), previously smoked (> 100 cigarettes/session, currently not smoking) and current smoker (> 100 cigarettes/session, either on some days or every day) [15]. Drinking status was categorized as non-drinkers (< 12 drinks in life), ever drinking in the last year (alcohol or 12 drinks in life, currently not drinking), mild/moderate drinkers (over the past year: females, once/day or less; males, twice/day or less), heavy drinkers (over the past year: females, more than once/day; males, > twice/day) [16]. Medical history and medication use were collected via family interviews and mobile examination centers using standardized questionnaires. The specific details for collecting these covariates can be obtained from the NHANES Laboratory/Medical Technician Procedure Manual [13].

### Statistical analysis

Appropriate weighting (MEC2yr) was conducted in the statistical analysis. In population baseline characteristics, continuous variables were expressed as weighted means (standard errors) and categorical variables as unweighted counts (weighted %). Hazard ratios (HRs) and 95% confidence intervals (CIs) of ePWV with all-cause and CVD mortality were assessed using survey-weighted cox regression models. As most of the data were skewed, the Mann-Whitney test was used to compare the two groups for continuous variables and the chi-squared test was used to compare the categorical variables. From baseline characteristics, confounders were selected according to their association with the outcome of interest or a change in the effect estimate of > 10% [17].

Additional file 1: Table S1 depicts the variables with a contribution of > 10% to each result. Meanwhile, for the missing data, we obtained five data sets by multiple imputations, and the pooled multivariate cox regression results were regarded as sensitivity analysis and the results were shown in Additional file 1: Table S2. Regarding the models in this study, we eventually made the following adjustments. For model 1 in all-cause and CVD mortality, we adjusted age, race and gender. For model 2 in all-cause mortality, we adjusted age, gender, race, educational level, marital status, poverty income ratio (PIR), waist, hemoglobin (Hb), HbA1c, fasting plasma glucose (FPG), alanine aminotransferase (ALT), tuberculosis (TB), creatinine, low-density lipoproteins (LDL), C-reactive protein (CRP), osteoporosis, chronic kidney disease (CKD), arthritis, CVD, diabetes mellitus (DM), hyperlipidemia, hypertension, antihypertensive medication, diabetes medications, alcohol use and smoke. For model 2 in CVD mortality, we adjusted age, race, educational level, marital status, BMI, PIR, waist, Hb, HbA1c, FPG, ALT, aspartate aminotransferase (AST), TB, creatinine, low-density lipoproteins (LDL), osteoporosis, CKD, arthritis, CVD, DM, hyperlipidemia, hypertension, antihypertensive medication, diabetes medications, alcohol use and smoke.

Subgroup analysis was conducted by following demographic covariates and CVD-risk factors including sex (male, female), age (< 60 years and ≥ 60 years), BMI (30–34.9,35–39.9, ≥ 40 kg/m^2^), race (non-Hispanic white people, non-Hispanic Black people, Mexican Americans, etc.), history of CVD (no/yes), and history of hypertension (no/yes), history of DM (no/yes), history of Asthma (no/yes), history of hyperlipidemia (no/yes), history of Arthritis (no/yes), history of Osteoporosis (no/yes), history of CKD (no/yes), and *P*-values for interaction were obtained. In addition, receiver operating characteristic (ROC) curve was used to assess the predictive value of ePWV for all-cause and CVD mortality. Furthermore, a generalized additive model was used to evaluate the association between ePWV and the risk of mortality [18], and *P*-values for non-linear regression were obtained using log-likelihood ratio tests. If a non-linear association was observed, a two-piecewise linear regression model was used to calculate the inflection point where the ratio of ePWV to mortality significantly changes in the smooth curve [19].

All statistical analyses were performed by R software (Version 4.2.1, http://www.R-project.org, The R Foundation) and EmpowerStats (Version 4.2.0, www.R-project.org, X&Y Solutions, Inc., Boston, MA). *P* < 0.05 was considered statistically significant.

## Results

### Baseline characteristics in the study

Data were collected from a total of 8315 participants with obesity, and those data represented 64,916,705 individuals. After a median follow-up of 101 months (interquartile range 75 − 134 months), 833 all-cause mortality deaths and 240 CVD deaths occurred.

The median level of ePWV was 7.87 m/s, and the low-ePWV group and the high-ePWV group included 4175 and 4158 participants, respectively. The specific results of survey-weighted baseline characteristics with high ePWV and low ePWV levels in participants with obesity are indicated in Table 1.

**Table 1:** Survey-weighted baseline characteristics of obese participants (representing 64,916,705 individuals) stratified by ePWV median levels

Variables	Low ePWV (4.78–7.87 m/s) n = 4157	High ePWV (7.87–16.26 m/s) n = 4158	P-value	Variables	Low ePWV (4.78–7.87 m/s) n = 4157	High ePWV (7.87–16.26 m/s) n = 4158	P-value
Represented size	35,722,926	29,193,779		No	2748 (98.61)	3045 (92.58)	
BMI (kg/m^2^)	35.72 (0.12)	35.88 (0.13)	0.162	Yes	42 (1.39)	265 (7.42)	
PIR	2.70 (0.05)	3.05 (0.05)	< 0.001	Arthritis			< 0.001
Waist (cm)	112.71 (0.29)	115.64 (0.30)	< 0.001	No	3476 (83.15)	2151 (52.65)	
HB (g/dL)	14.32 (0.04)	14.28 (0.04)	< 0.001	Yes	677 (16.85)	1996 (47.35)	
HBA1C (%)	5.61 (0.02)	6.09 (0.03)	< 0.001	CKD			< 0.001
FPG (mg/dL)	106.67 (0.74)	120.46 (1.62)	< 0.001	No	3547 (91.52)	2745 (74.66)	
AST (U/L)	26.02 (0.23)	26.60 (0.34)	0.017	Yes	380 (8.48)	1224 (26.34)	
ALT (U/L)	30.53 (0.34)	27.29 (0.40)	< 0.001	DM			< 0.001
TB (μmol/L)	11.50 (0.12)	12.06 (0.13)	< 0.001	No	3396 (88.34)	2584 (68.05)	
Creatinine(μmol/L)	75.57 (0.47)	82.69 (0.56)	< 0.001	Yes	561 (11.66)	1574 (31.95)	
HDL (mmol/L)	1.18 (0.01)	1.27 (0.01)	< 0.001	CVD			< 0.001
TC (mmol/L)	5.05 (0.02)	5.14 (0.03)	0.011	No	3397 (96.41)	2584 (82.89)	
LDL (mmol/L)	3.06 (0.03)	2.98 (0.03)	< 0.001	Yes	159 (3.59)	789 (17.11)	
TG (mmol/L)	1.66 (0.04)	1.86 (0.05)	< 0.001	Hypertension			< 0.001
CRP (mg/dL)	0.60 (0.02)	0.57 (0.02)	0.316	No	2977 (71.63)	998 (27.32)	
Age			< 0.001	Yes	1180 (28.37)	3160 (72.68)	
< 60 years	4133 (99.45)	1745 (51.61)		Hyperlipidemia			< 0.001
≥ 60 years	24 (0.55)	2413 (48.39)		No	1107 (25.06)	688 (15.24)	
Gender			0.529	Yes	3050 (74.94)	3470 (84.76)	
Women	2293 (51.90)	2265 (52.89)		Antihypertensive medication			< 0.001
Men	1864 (48.10)	1893 (47.11)		No	4000 (96.10)	3499 (85.31)	
Race			< 0.001	Yes	157 (3.90)	656 (14.69)	
Non-Hispanic white	1591 (59.61)	1736 (70.27)		Diabetes medications			< 0.001
Non-Hispanic black	1079 (15.76)	1223 (15.29)		No	3846 (93.21)	3113 (79.13)	
Mexican American	842 (13.04)	681(6.94)		Yes	311 (6.79)	1042 (20.87)	
Other races	645 (11.59)	518 (7.50)		Alcohol user			< 0.001
Education Levels			< 0.001	Never	501 (9.82)	636 (12.46)	
Less than 9th grade	315 (4.79)	604 (7.42)		Former	552 (13.02)	1081(22.74)	
9-11th grade/high school grade or equivalent	1691 (37.50)	1725 (39.59)		Mild/moderate	1034 (27.26)	1229 (34.12)	
College graduate or above	2146 (57.71)	1827 (52.99)		Heavy	1714 (42.76)	958 (25.26)	
Marital Status			< 0.001	Refused/Don't know	356 (7.14)	254 (5.41)	
Never married	1128 (24.23)	363 (8.28)		Smoke status			< 0.001
Married	2011 (53.09)	2286 (60.93)		Never	2546 (59.81)	2176 (51.76)	
Separated	163 (2.76)	156 (2.41)		Former	642 (17.63)	1376 (34.24)	
Divorced	362 (9.14)	640 (14.92)		Current	967 (22.56)	603 (14.0)	
Widowed	36 (0.63)	572 (10.28)					
Living with partner/others	457 (10.13)	141 (3.19)					
Asthma			0.003				
No	3404 (81.86)	3510 (84.63)					
Yes	747 (18.14)	646 (15.37)					
Osteoporosis			< 0.001				

Continuous variables are expressed as weighted mean (Standard error, SE). Categorical variables are expressed as counts (weighted %)

*ePWV* estimated pulse wave velocity, *CVD* cardiovascular diseases, *PIR* Poverty income ratio, *CKD* Chronic kidney disease, *BMI* body mass index, *HB* hemoglobin, *FPG* fasting plasma glucose, *TB* Total bilirubin, *TC* Total cholesterol, *LDL* low-density lipoprotein cholesterol, *TG* triglycerides, *HDL* high-density lipoprotein cholesterol, *ALT* alanine aminotransferase, *AST* aspartate aminotransferase, *HbA1C* glycated hemoglobinA1c

### Univariate and multivariate cox regression analyses of the association between ePWV and all-cause and CVD mortality

Table 2 depicts the association between ePWV levels and the risk of all-cause and CVD mortality using weighted univariable and multivariable cox regression analyses in participants with obesity. During the follow-up of this investigation, there were 722 deaths in the high-ePWV group and 111 in the low-ePWV group, respectively. We further analyzed the effect of ePWV levels on all-cause and CVD mortality using univariate cox regression, and the crude model demonstrated that high ePWV level (7.87 − 16.26 m/s) was an independent risk factor in both all-cause mortality (HR = 6.36, 95% CI 4.92 − 8.22, *P* < 0.001) and CVD mortality (HR = 8.7, 95% CI 4.57 − 16.55, *P* < 0.001). In addition, every 1 m/s-increase in ePWV increased the risk of both all-cause mortality (HR = 1.65, 95% CI 1.59 − 1.72, *P* < 0.001) and CVD mortality (HR = 1.77, 95% CI 1.61 − 1.85, *P* < 0.001).

**Table 2:** Weighted univariate and multivariate Cox regression to assess the association between ePWV levels and the risk of all-cause and cardiovascular disease mortality in obese participants

	Low ePWV (4.78–7.87 m/s)	High ePWV (7.87–16.26 m/s)	p-value	Every 1 m/s increase in ePWV	p-value
All-cause mortality					
Number of deaths	111	722		833	
Crude model	Ref	6.36 (4.92–8.22)	< 0.001	1.65 (1.59–1.72)	< 0.001
Model 1*	Ref	2.64 (1.83–3.80)	< 0.001	1.47 (1.38–1.57)	< 0.001
Model 2*	Ref	2.25 (1.12–4.90)	0.024	2.23 (1.26–3.93)	0.006
CVD Mortality					
Number of deaths	24	216		240	
Crude model	Ref	8.70 (4.57–16.55)	< 0.001	1.77 (1.61–1.85)	< 0.001
Model 1*	Ref	2.60 (1.12–6.02)	0.026	1.43 (1.29–1.61)	< 0.001
Model 2 †	Ref	6.76 (1.09–41.92)	0.040	1.44 (1.14–1.82)	0.003

Model 1* adjusted age, race, gender

Model 2*Age, Gender, Race, Education levels, Marital Status, PIR, Waist, HB, HBA1c, FPG, ALT, TB, Creatinine, LDL, CRP, Osteoporosis, CKD, Arthritis, CVD, DM, Hyperlipidemia, Hypertension, Antihypertensive medication, Diabetes medications, Alcohol use, Smoke

Model 2 † Age, Race, Education levels, Marital Status, BMI, PIR, Waist, HB, HBA1c, FPG, ALT, AST, TB, Creatinine, LDL, Osteoporosis, CKD, Arthritis, CVD, DM, Hyperlipidemia, Hypertension, Antihypertensive medication, Diabetes medications, Alcohol use, Smoke

Next, multivariate cox regression analysis was conducted to evaluate ePWV levels and the risk of all-cause and CVD mortality in participants with obesity (Table 2). Three models were constructed after adjusting for different covariates. In all these models, high ePWV level was an independent risk factor for all-cause and CVD mortality. Besides, every 1 m/s-increase in ePWV was associated with a 123% and 44% increase in the risk of all-cause mortality (HR = 2.23, 95% CI 1.26 − 3.93, *P* = 0.006) and CVD mortality (HR = 1.44, 95% CI 1.14 − 1.82, *P* = 0.003) in the corresponding model 2 with maximum adjusted covariates, respectively.

### Subgroup analysis

**Fig. 1:**
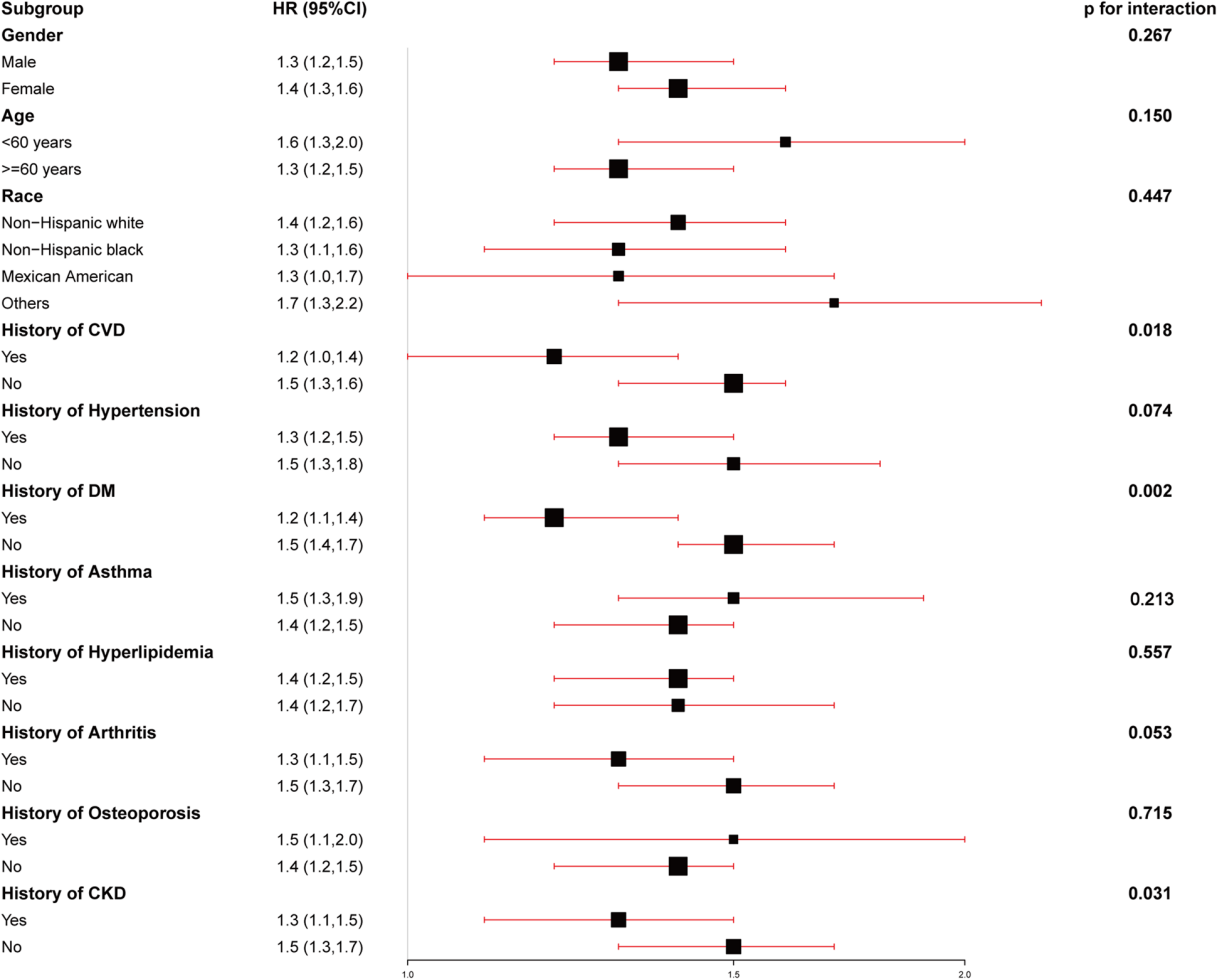
Subgroups analyses of the correlation between clinical characteristics and all-cause mortality. The model was adjusted for age, gender, body mass index (BMI), race, education levels, marital status, poverty income ratio (PIR), waist, hemoglobin (Hb), HbA1c, fasting plasma glucose (FPG), alanine aminotransferase (ALT), tuberculosis (TB), creatinine, low-density lipoproteins (LDL), C-reactive protein (CRP), osteoporosis, chronic kidney disease (CKD), arthritis, cardiovascular disease (CVD), diabetes mellitus (DM), hyperlipidemia, hypertension, antihypertensive medication, diabetes medications, alcohol use and smoke

**Fig. 2:**
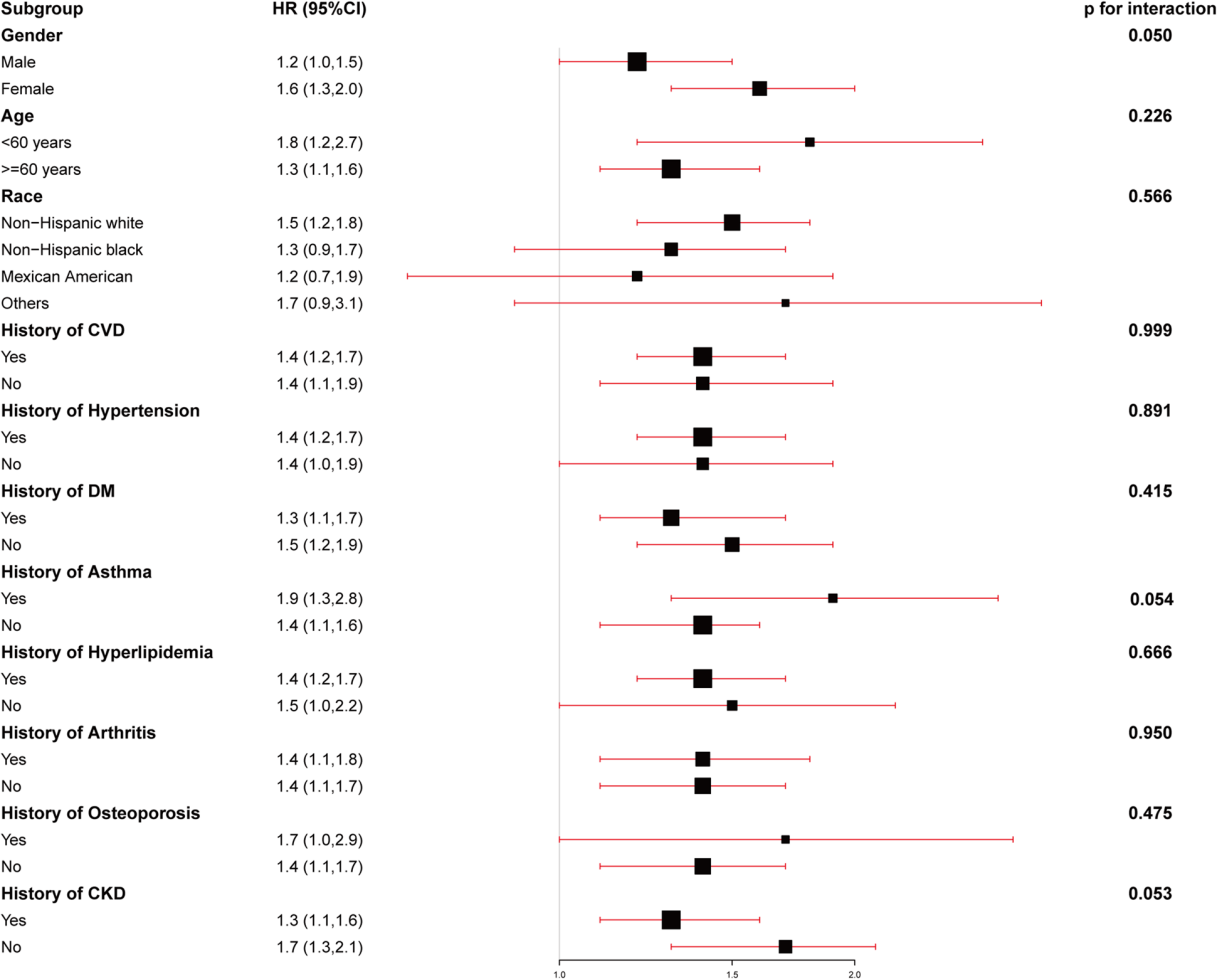
Subgroups analyses of the correlation between clinical characteristics and cardiovascular disease (CVD) mortality. The model was adjusted for age, body mass index (BMI), race, education levels, marital status, poverty income ratio (PIR), waist, hemoglobin (Hb), HbA1c, fasting plasma glucose (FPG), alanine aminotransferase (ALT), aspartate aminotransferase (AST), tuberculosis (TB), creatinine, low-density lipoproteins (LDL), osteoporosis, chronic kidney disease (CKD), arthritis, cardiovascular disease (CVD), diabetes mellitus (DM), hyperlipidemia, hypertension, antihypertensive medication, diabetes medications, alcohol use and smoke

As indicated in Fig. 1 and 2, subgroup analyses were performed to assess the association of ePWV with all-cause and CVD mortality based on the different clinical characteristics of the participants. In most subgroups, the results for ePWV and all-cause mortality were consistent (*P* > 0.05, Fig. 1). However, the relationship between ePWV and all-cause mortality was slightly different in the subgroups with histories of CVD (*P* = 0.018 for interaction), DM (*P* = 0.002 for interaction), or CKD (*P* = 0.031 for interaction). In addition, the results for ePWV and CVD mortality were consistent in all CVD mortality subgroups (all *P* for interaction were > 0.05, Fig. 2).

### Diagnostic value of ePWV

**Fig. 3:**
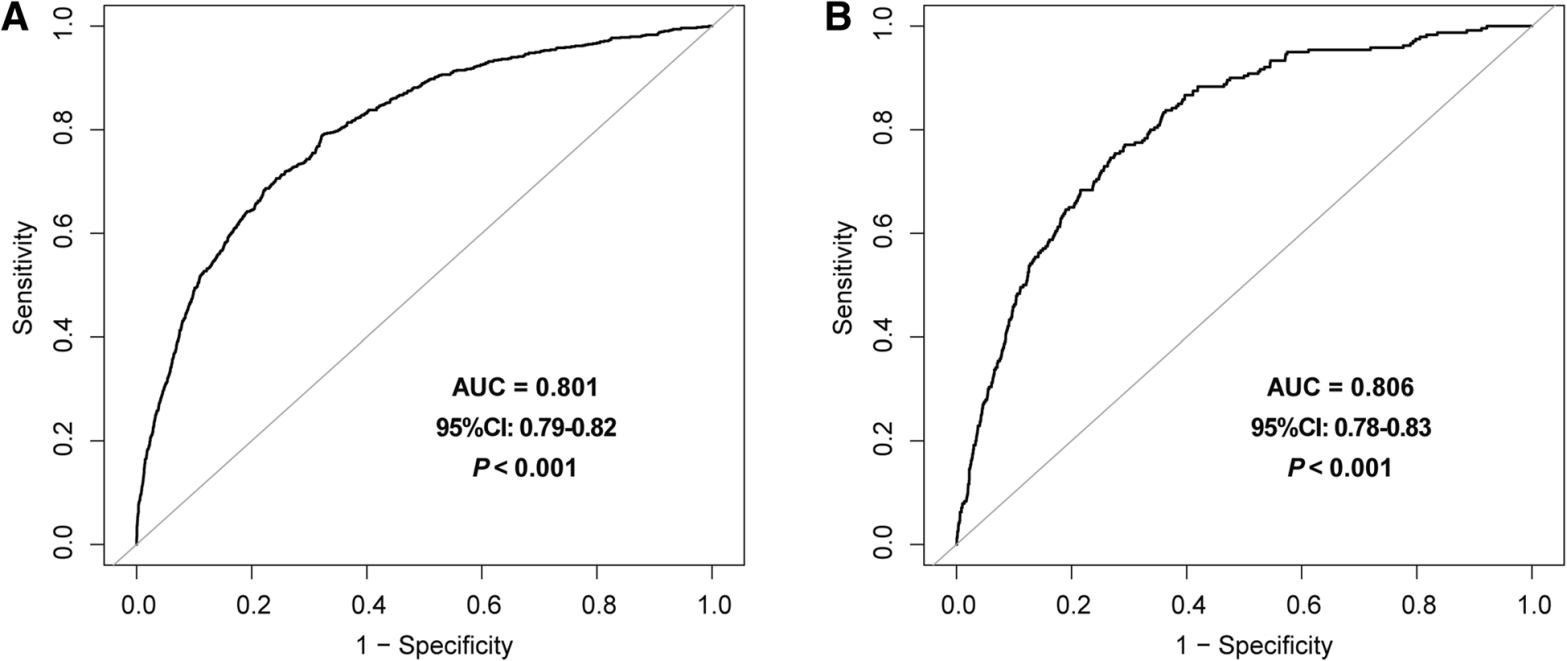
Receiver operating characteristics (ROC) curves for ePWV as a predictor of all-cause mortality **A** and CVD mortality **B**

ROC analysis was constructed to evaluated the diagnostic value of ePWV in all-cause mortality and CVD mortality. The results demonstrated that ePWV had a certain accuracy in predicting all-cause mortality (AUC = 0.801, 95% CI 0.79–0.82, *P* < 0.001) and CVD mortality (AUC = 0.806, 95% CI 78–0.83, *P* < 0.001). The ePWV cutoff point value for the group with all-cause mortality, where the sensitivity and specificity were highest (0.79 and 0.68, respectively) was 8.7 m/s. And for the group with CVD mortality, where the sensitivity and specificity were highest (0.75 and 0.73, respectively) was 9.3 m/s (Fig. 3A and B).

### Two-piecewise linear regression model to assess the association between ePWV and the risk of all-cause and CVD mortality

As indicated in Table 3 and Additional file 1: Table S3, a two-piecewise linear regression model was used to assess the association between the risk of ePWV and all-cause and CVD mortality based on respective model 2. ePWV had a non-linear association with the risk of both all-cause mortality and CVD mortality (non-linear *P* < 0.001) with two inflection points for each group (6.7 m/s and 8.7 m/s for all-cause mortality; 7.2 m/s and 8.5 m/s for CVD mortality). A positive correlation was observed between ePWV and all-cause mortality when ePWV > 6.7 m/s (*P* < 0.001), and the ePWV was positively correlated with CVD mortality when ePWV > 7.2 m/s (*P* < 0.001, Table 3).

**Table 3:** The results of two-piecewise linear regression model for ePWV and the risk of all-cause and CVD mortality in obese participants

Outcome	Inflection-point of ePWV (m/s)	HR	95% CI	*P*-value
All-cause Mortality				
	< 6.7	0.5	0.1–2.0	0.314
	6.7–8.7	3.2	1.9–5.7	< 0.001
	≥ 8.7	1.3	1.2–1.6	< 0.001
CVD Mortality	< 7.2	0.5	0.1–2.0	0.245
	7.2–8.5	16.1	2.0–126.8	0.008
	≥ 8.5	1.3	1.1–1.6	< 0.001

HRs have been fully adjusted for confounders, which are the same as the variables adjusted for Model 2 in Table2 (Model 2* for all-cause mortality, Model 2 † for CVD mortality, respectively)

**Fig. 4:**
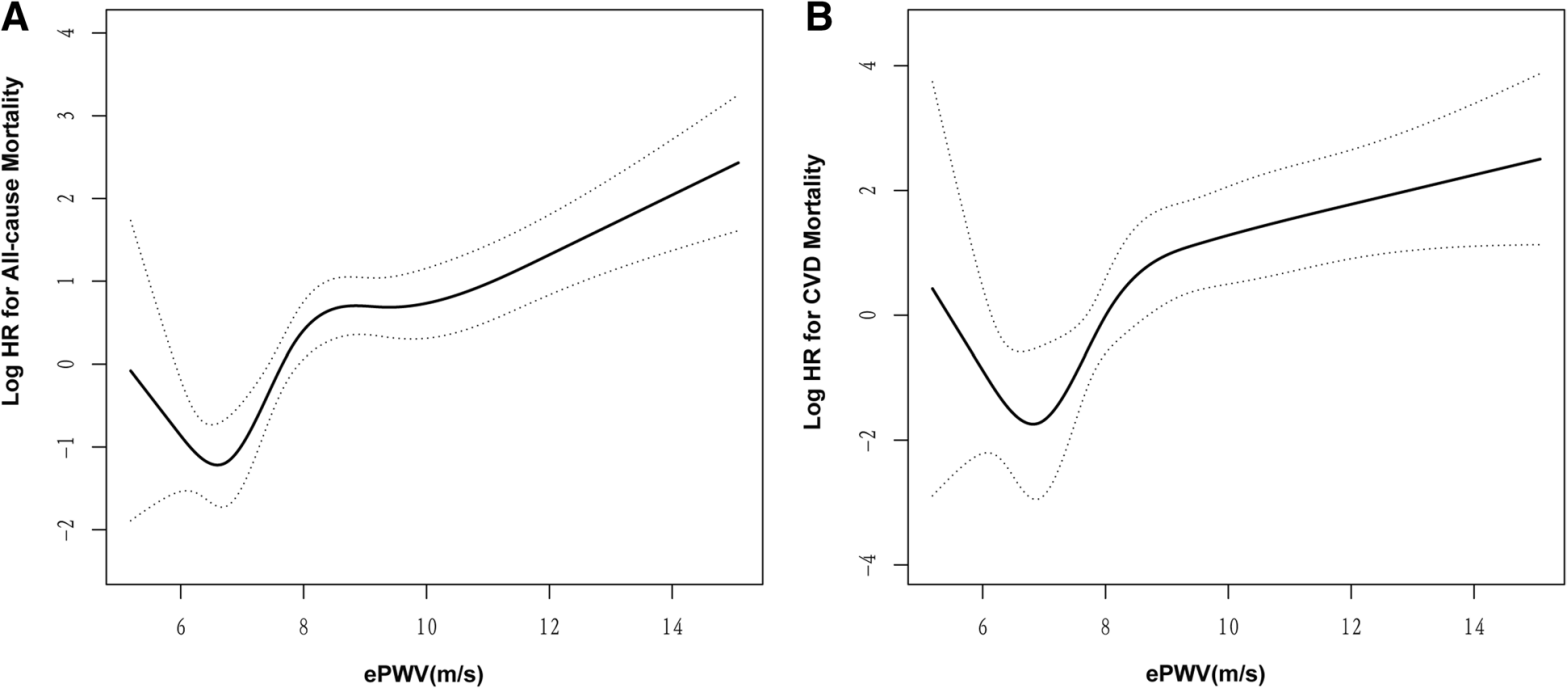
Visualization curves of two-piecewise linear regression model for ePWV and the risk for all-cause mortality (**A**) and CVD mortality (**B**). The solid line and long dashed line represent the estimated odds ratio and its 95% confidence interval. ePWV: estimated pulse wave velocity

Meanwhile, a steeper growth curve was observed in all-cause mortality and CVD mortality when ePWV values were 6.7 − 8.7 m/s and 7.2 − 8.5 m/s, respectively. In this interval, every 1 m/s-increase in ePWV was associated with a 3.2-fold (95% CI 1.9 − 5.7, *P* < 0.001) and 16.1-fold (95% CI 2.0 − 126.8, *P* = 0.008) increase in the risk of all-cause mortality and CVD mortality. This demonstrated that the all-cause mortality increased more readily when ePWV values fell within this interval. Afterwards, smooth growth curves were observed when ePWV > 8.7 m/s (HR = 1.3, 95% CI 1.2 − 1.6, *P* < 0.001) and 8.5 m/s (HR = 1.3, 95% CI 1.1 − 1.6, *P* < 0.001) for all-cause mortality and CVD mortality, respectively (Table 3). The results for all-cause mortality and CVD mortality are demonstrated in Figs. 4A and B.

Finally, we explored the effect of ePWV on all-cause mortality and CVD mortality in different BMI, and the results were shown in Additional file 1: Table S3. For the participants with a BMI in 30–40 kg/m^2^, a steeper growth curve was observed in all-cause mortality and CVD mortality when ePWV values were below 8.45 m/s (HR = 2.77, 95% CI 1.7 − 4.5, *P* < 0.001) and 8.73 m/s (HR = 5.05, 95% CI 1.49 − 17.18, *P* < 0.001), respectively. And for the participants with a BMI ≥ 40 kg/m^2^, a similar steeper curve was found when ePWV < 11.34 m/s for CVD mortality. However, there was no significant correlation between ePWV and all-cause mortality and CVD mortality when ePWV > 8.45 m/s (HR = 1.41, 95% CI 1.00 − 1.99, *P* = 0.05) and 11.34 m/s (HR = 0.88, 95% CI 0.17 − 4.57, *P* = 0.883).

## Discussion

This study was the first large retrospective observational study based on individuals with obesity to examine the relationship between ePWV levels and all-cause and CVD mortality. Our findings can be summarized as follows, (1) ePWV was an independent risk factor for mortality in populations with obesity; (2) higher ePWV was significantly associated with an increased risk of both all-cause and CVD mortality; (3) ePWV is non-linearly related to the risk of all-cause and CVD mortality. Each 1 m/s-increase in ePWV was associated with a 123% and 44% increase in the risk of all-cause mortality and CVD mortality, respectively.

Subgroup analysis indicated that the association between ePWV and all-cause mortality was differed by the histories of CVD, DM and CKD, the increase of ePWV may pose an increased risk of all-cause mortality in the participants without a history of CVD, DM or CKD.The underlying mechanism is still unclear. ePWV is an indicator to assess the degree of arterial stiffness. Increased arterial stiffness is an important factor in the development and progression of chronic diseases including CVD, DM and CKD, hypertension, asthma, hyperlipidemia, arthritis, osteoporosis, etc. [20–22]. The caused may include the following aspects. Firstly, arterial stiffness generates a dramatic increase in blood pressure, leading to cerebrovascular accidents, aneurysms and other accidents [23]. Secondly, arterial stiffness affects blood circulation, reducing the delivery of oxygen and nutrients and leading to impaired organ function, thereby increasing the risk of all-cause mortality [24]. However, the interweaving of multiple factors may weaken the relationship between ePWV and all-cause mortality. Besides, patients with a history of CVD, DM and CKD, early intervention and treatment are often effective in reducing the risk of mortality [25, 26] which further weakens the association between ePWV and all-cause mortality.

Many studies have considered ePWV > 10 m/s an important risk factor for the development of related diseases [27–29]. However, Further analysis is required because this division based solely on median value cannot capture the overall trend in predicting disease risk. In this study, based on the median ePWV value, we demonstrated that ePWV > 7.87 m/s is a significant risk factor for all-cause and CVD mortality. However, on further analysis using the two-piecewise linear regression model for ePWV and the risk of all-cause and CVD mortality, we demonstrated that the threshold value for distinguishing the ePWV hazard effect was 6.7 m/s for all-cause mortality and 7.2 m/s for CVD mortality, respectively. Since they are all below the median value, some participants with ePWV values between the median (7.87 m/s) and threshold values (6.7 m/s or 7.2 m/s) may be categorised as belonging to the low-risk group.

Obesity is correlated with increased arterial stiffness [30, 31]. The structural and functional changes of the intima, middle layer and outer layer of the vasculature are the main causes of arterial stiffness in patients with obesity, which can be regulated by plasma factors such as aldosterone and insulin [32]. Besides, abnormal distribution, immune cell dysfunction and extracellular matrix remodeling are all important factors of arterial stiffness in patients with obesity [33–35]. Meanwhile, metabolic dysfunction increases insulin and aldosterone levels, activating the endothelial Na^+^ channel [32]. This subsequently causes an impaired release of nitric oxide (NO) produced by vascular endothelial cells, restricted diastole of vascular smooth muscle cells, stimulation of tissue remodeling and the development of fibrosis and thus vascular stiffness [36].

In addition, adipokines such as adiponectin and leptin, secreted by adipocytes, are also closely associated with arterial stiffness [37]. Plasma circulating adiponectin levels are inversely correlated with total body fat mass [38]. Adiponectin exerts anti-inflammatory and anti-fibrotic effects and enhances insulin sensitivity [39]. In addition, adiponectin reduces endothelial cell apoptosis [40], increases NO utilisation and improves vascular endothelial dysfunction [41]. Previous studies have revealed that low adiponectin levels are a risk factor for arterial stiffness [42, 43]. Plasma leptin levels increase linearly with increasing body weight, and leptin induces hypertension and endothelial dysfunction using aldosterone-dependent methods [44]. A large cross-sectional study demonstrated a synergistic relationship between high plasma leptin and low adiponectin levels and the progression of arterial stiffness [45]. In addition, the results of a meta-analysis revealed that leptin was positively correlated with arterial stiffness [46].

Arterial stiffness has been reported to exhibit a correlation with the morbidity and mortality of hypertension and CVD [47, 48]. Although cf-PWV and baPWV are widely regarded as the gold standard for assessing arterial stiffness in a non-invasive manner to predict the risk of CVD [8, 9], they necessitate specialized knowledge and expensive instrumentation. Compared with cf-PWV, ePWV is a convenient and affordable indicator that combines age and BP to evaluate arterial stiffness. In recent years, ePWV has widely been used as an indicator to predict disease risk in various populations, including the general population, patients with stroke, and those with CVD [27, 28, 49]. Moreover, ePWV revealed a better performance than the Framingham risk score (FRS) in predicting CVD (*P* = 0.044) and overall mortality (*P* < 0.001); however, no significant difference was observed between baPWV and FRS (*P* > 0.05 for both) [29]. Therefore, ePWV is suitable for widespread screening and self-monitoring usage in the population.

In this study, we discovered the steeper growth curve for participants with ePWVs of 6.7 − 8.7 m/s for all-cause mortality and 6.2 − 8.5 m/s for CVD mortality, respectively. This indicates that until ePWV reaches a high threshold, the risk of all-cause mortality increases more quickly, and high BP is a distinctive feature of arterial stiffness [50], which is associated with reduced arterial compliance and causes increased myocardial fiber contractility and left ventricular load and ventricular diastolic dysfunction [51, 52]. Heart failure is caused by sudden, severe BP swings, which increase the risk of patient death [53]. This may explain the sharp rise in patient mortality before the specific threshold at which ePWV increased. The ventricles become tolerant owing to the gradual rise in BP, which delays the rate of mortality. Another possible reason could be that participants with higher ePWV were more conscious of their health status and thus sought medical assistance and made lifestyle improvements. This caused a slightly downward trend in all-cause mortality and CVD mortality.

## Strengths and limitations

This study was a pioneering assessment of the predictive value of ePWV on all-cause and CVD mortality in a population with obesity. We demonstrated the dynamic model of ePWV affecting all-cause mortality and CVD mortality, which can be used to assess the prognosis of populations with obesity. However, our study had some limitations. First, our results are only partially representative of this region as the population used in this survey is primarily from parts of the United States and cannot be representative of all regions of the world. Second, Asian-Americans and native Americans have different diagnostic criteria for obesity, which may cause some bias into the results. Last, this study did not collect comprehensive information on participants’ drugs or other chronic diseases, which might have partially affected the findings.

## Conclusion

ePWV was an independent risk factor for mortality in the populations with obesity. High ePWV levels were associated with increased all-cause mortality and cardiovascular mortality. Therefore, ePWV can be considered a novel biomarker to assess the risk of mortality in patients with obesity.

## Supplementary Information

**Additional file 1: Table S1.** Selected covariates. **Table S2.** Survey-weighted multivariate Cox regression performed to assess the ePWV levels and the risk of all-cause and CVD mortality after multiple imputation of 5 data sets. Table S3. The results of two-piecewise linear regression model for ePWV and the risk of all-cause and CVD mortality in obese participants with different BMI.

## Data Availability

Elaborated information is available at https://wwwn.cdc.gov/Nchs/Nhanes/. All the data were also available from the corresponding authors for reasonable request.

## Abbreviations

ePWV: Estimated pulse wave velocity
CVD: Cardiovascular disease
PWV: Pulse wave velocity
cfPWV: Carotid-femoral PWV
baPWV: Brachial-ankle PWV
MB: Mean blood pressure (MBP)
NHANES: National Health and Nutrition Examination Survey
HRs: Hazard ratios
CI: Confidence intervals
PIR: Poverty income ratio
Hb: Waist, hemoglobin
FPG: Fasting plasma glucose
ALT: Alanine aminotransferase
LDL: Creatinine, low-density lipoproteins
CRP: C-reactive protein
CKD: Chronic kidney disease
ST: Aspartate aminotransferase
