# Supplementary material for: Predictive value of estimated pulse wave velocity for cardiovascular and all-cause mortality in individuals with obesity
Source: Diabetol Metab Syndr. 2023 Mar 9;15:40. doi: 10.1186/s13098-023-01011-2 (PMC9997019; doi:10.1186/s13098-023-01011-2)
Supplement: Supplementary file 1 — Additional file 1: Table S1. Selected covariates. Table S2. Survey-weighted multivariate Cox regression performed to assess the ePWV levels and the risk of all-cause and CVD mortality after multiple imputation of 5 data sets. Table S3. The results of two-piecewise linear regression model for ePWV and the risk of all-cause and CVD mortality in obese participants with different BMI. [file 13098_2023_1011_MOESM1_ESM.docx]

**Table S1. Selected covariates**

| **Y** | **X** | **Selected based on criteria 1** | **Selected based on criteria 2** |
| --- | --- | --- | --- |
| All-cause  Mortality | ePWV  Dichotomous | Age, CKD, CVD | Age, Gender, Race, Education levels, Marital Status, PIR, Waist, HB, HBA1c, FPG, ALT, TB, Creatinine, LDL, CRP, Osteoporosis, CKD, Arthritis, CVD, DM, Hyperlipidemia, Hypertension, Antihypertensive medication, Diabetes medications, Alcohol use, Smoke |
| CVD Mortality | ePWV Dichotomous | Age, CKD, Hypertension | Age, Race, Education levels, Marital Status, BMI, PIR, Waist, HB, HBA1c, FPG, ALT, AST, TB, Creatinine, LDL, Osteoporosis, CKD, Arthritis, CVD, DM, Hyperlipidemia, Hypertension, Antihypertensive medication, Diabetes medications, Alcohol use, Smoke |

**Notes:**

**Criteria 1：add the covariate to basic model or remove it from full model, change X coefficient. >10%.
Criteria 2：effect of criterion 1 or covariates on the p-value of the regression coefficient of Y < 0.1**

| **Table S2. Survey-weighted multivariate Cox regression** **performed to assess the ePWV levels and the risk of all-cause and CVD mortality after multiple imputation of 5 data sets.** | | | | | | |
| --- | --- | --- | --- | --- | --- | --- |
| **Low vs. High** | Multiple Imputation1 | Multiple Imputation 2 | Multiple Imputation 3 | Multiple Imputation 4 | Multiple Imputation 5 | **Pooled results** |
| **All-cause mortality** | 3.95 (1.56 to 3.75) | 3.91 (1.54 to 3.70) | 3.95 (1.56 to 3.78) | 3.90 (1.54 to 3.72) | 3.93 (1.56 to 3.80) | **3.93 (2.53 to 6.11)** |
| **CVD mortality** | 3.01 (1.13 to 8.02) | 3.05 (1.15 to 8.09) | 3.08 (1.15 to 8.22) | 3.05 (1.16 to 8.07) | 3.14 (1.17 to 8.45) | **3.07 (1.15 to 8.17)** |
| **Every 1m/s ePWV increase** |  |  |  |  |  |  |
| **All-cause mortality** | 1.38 (1.25 to 1.52) | 1.38 (1.25 to 1.52) | 1.37 (1.25 to 1.51) | 1.38 (1.25 to 1.52) | 1.38 (1.25 to 1.52) | **1.38 (1.25 to 1.52)** |
| **CVD mortality** | 1.35 (1.19 to 1.53) | 1.35 (1.19 to 1.53) | 1.36 (1.20 to 1.54) | 1.36 (1.21 to 1.55) | 1.36 (1.20 to 1.55) | **1.36 (1.20 to 1.54)** |

**HRs have been fully adjusted as described in the Table 2.**

| **Table S3. The results of two-piecewise linear regression model for ePWV and the risk of all-cause and CVD mortality in obese participants with different BMI.** | | | | |
| --- | --- | --- | --- | --- |
| **Outcome** | **Inflection-point of ePWV (m/s)** | **HR** | **95% CI** | ***P*-value** |
| **All-cause**  **Mortality** |  |  |  |  |
| **BMI 30-40** | ＜8.45 | 2.77 | 1.7-4.5 | ＜0.001 |
|  | ≥8.45 | 1.35 | 1.2-1.6 | ＜0.001 |
| **BMI >40** | - | 1.41 | 1.00-1.99 | 0.05 |
| **CVD Mortality** |  |  |  |  |
| **BMI 30-40** | ＜8.73 | 5.05 | 1.49-17.18 | 0.01 |
|  | ≥8.73 | 1.33 | 1.05-1.67 | 0.017 |
| **BMI >40** | ＜11.34 | 3.32 | 1.18-9.35 | 0.023 |
|  | ≥11.34 | 0.88 | 0.17-4.57 | 0.883 |

**HRs have been fully adjusted for confounders, which are the same as the variables adjusted for Model 2 in Table2 (Model 2* for all-cause mortality, Model 2 † for CVD mortality, respectively)**
